# Attitudes and (Mis)information About Cognitive Behavioral Therapy on TikTok: An Analysis of Video Content

**DOI:** 10.2196/45571

**Published:** 2023-03-13

**Authors:** Lorenzo Lorenzo-Luaces, Clare Dierckman, Sydney Adams

**Affiliations:** 1 Psychological and Brain Sciences Indiana University-Bloomington Bloomington, IN United States; 2 Luddy School of Informatics, Computing, and Engineering Indiana University-Bloomington Bloomington, IN United States

**Keywords:** social media, cognitive behavioral therapy, misinformation, public health, mental health, TikTok, psychotherapy, content analysis, therapist, misinformation, online health information

## Introduction

Cognitive behavioral therapy (CBT) is the most widely studied form of psychotherapy [1]. CBT is efficacious for internalizing disorders like depression, anxiety, and posttraumatic stress disorder (PTSD) [2]. For some clinical problems (eg, insomnia, obsessive-compulsive disorder), CBT is the only empirically supported treatment. We explored the social media platform TikTok to understand public attitudes and information about CBT. This work is important because individuals consume health-related information on social media, and their attitudes predict whether individuals will pursue treatment [3]. Prior work suggests individuals discuss their experiences with antidepressants on social media [4].

## Methods

We searched for “cognitive behavioral therapy” on TikTok (September 13, 2022) and viewed the first 50 videos to identify potential themes, which informed a codebook for thematic analysis [5]. The codebook, data, and analyses are available online [6]. Trained raters (CD and SA) identified the top 200 CBT videos that were produced again by searching on November 18, 2022. We chose 200 as a number that could be reasonably coded by two raters. The principal investigator (LLL) resolved disagreements. We rated the tone of the TikTok post (positive/neutral/mixed/negative); whether the poster alleged to be a mental health professional, distinguishing between three categories, (1) nonprofessionals (eg, former patient), (2) mental health coaches, and (3) mental health professionals, not coaches (eg, PhD level); and whether the poster alleged to have undergone CBT. We also recorded metadata (ie, the number of views, likes, and comments). For videos containing negative content, we rated the nature of the critiques. We tested for differences in continuous distributions using Wilcoxon rank sum test and in categorical variables using Pearson chi-square test or Fisher exact test (when n<5).

## Results

From our search, 32 videos were not CBT related. Of the 168 CBT videos, most were posted by individuals that claimed to be mental health professionals (Table 1). Most were positive (n=124, 73.8%). A minority described factual information (ie, neutral: n=6, 3.6%) or were mixed (n=7, 4.2%), claiming CBT had positive and negative features. About one-fifth were entirely negative (n=31, 18.5%). We grouped videos into a category indicating the presence of any negativity (ie, mixed or negative: n=38, 22.6%) versus its absence.

Individuals who claimed to have undergone CBT were more likely to express negative views about CBT than those who did not report this (odds ratio [OR] 3.77, 95% CI 1.70-8.41). Additionally, videos with a mixed/negative tone had substantially more comments than videos with a positive/neutral tone (standardized median difference 0.68, 95% CI 0.23-1.13).

The most common critiques were that CBT is ineffective or invalidating (Table 2). Other concerns included CBT being ineffective/harmful for individuals reporting trauma and stressor-related disorders, neurodevelopmental disorders, or systematic oppression (eg, individuals from racial/ethnic minoritized groups).

**Table 1:** Characteristics of the top 168 TikTok videos about cognitive behavioral therapy, overall and by the tone of the video (negative/mixed vs positive/neutral).

Characteristic		Overall (N=168)	Positive/neutral (n=130)	Negative/mixed (n=38)	*P* value^a^	
**TikTok poster, n (%)**						.75
	Not identified as a professional (eg, former patient)	63 (37.5)	47 (36.2)	16 (42.1)		
	Self-identified as a mental health coach	32 (19.1)	26 (20.0)	6 (15.8)		
	Self-identified as a mental health professional, not coach (eg, PhD-level psychologist, psychiatrist)	73 (43.5)	57 (43.9)	16 (42.1)		
Underwent cognitive behavioral therapy, n (%)		37 (22.0)	21 (16.2)	16 (42.1)	<.001	
**Total number of views, median (IQR)**		5905 (2676-39,300)	5111 (1994-38,900)	7184 (3784-35,525)	.21	
	Unknown, n (%)	1 (0.6)	1 (0.8)	0 (0.0)		
Total number of likes, median (IQR)		469 (145-3019)	458 (110-2940)	560 (274-3010)	.14	
Total number of comments, median (IQR)		23 (6-70)	15 (4-57)	39 (18-162)	<.001	

^a^Pearson chi-square test or Wilcoxon rank sum test.

**Table 2:** Main critiques of CBT in TikToks (n=38) with negative content by the background of the TikTok creator.

Characteristic	Overall (n=38), n (%)	Not identified as a professional (eg, former patient; n=16), n (%)	Self-identified as a mental health coach (n=6), n (%)	Self-identified as a mental health professional, not coach (eg, PhD-level psychologist, psychiatrist; n=16), n (%)	*P* value^a^
Invalidation: CBT^b^ is invalidating (eg, a form of gaslighting)	19 (50)	9 (56)	2 (33)	8 (50)	.76
Trauma: CBT is generally ineffective or harmful for people with a history of trauma, PTSD^c^, or a trauma-related disorder	12 (32)	3 (19)	2 (33)	7 (44)	.37
Lack of efficacy/iatrogenic effects: In general, CBT is generally ineffective or harmful	21 (55)	8 (50)	3 (50)	10 (62)	.76
Neurodiversity: CBT is ineffective or harmful for individuals with neurodevelopmental disorders (eg, ADHD^d^, ASD^e^)	9 (24)	6 (38)	1 (17)	2 (12)	.28
Systematic oppression: CBT is harmful or ineffective for individuals experiencing systematic oppression (eg, racial/ethnic minorities, LGBTQ+^f^, or people with disabilities)	8 (21)	4 (25)	0 (0)	4 (25)	.65
Financial incentives: CBT is only practiced because it is reimbursed by insurance companies	3 (8)	0 (0)	1 (17)	2 (12)	.36

^a^Fisher exact test.

^b^CBT: cognitive behavioral therapy.

^c^PTSD: posttraumatic stress disorder.

^d^ADHD: attention-deficit/hyperactivity disorder.

^e^ASD: autism spectrum disorder.

^f^LGBTQ+: lesbian, gay, bisexual, transgender, queer.

## Discussion

Relative to the low rate of negative outcomes reported in treatment trials [7], negative content about CBT is common on TikTok and is focused on the efficacy of CBT and its suitability for specific subgroups of patients. Our findings should be interpreted with caution as our study was exploratory, and a number of variables relied on self-report. However, like prior work on antidepressants, our data suggest that individuals discuss rather negative aspects of mental health treatments on social media (eg, side effects) [4].

There are at least two interpretations of our findings. First, social media may offer a window into the quality of treatment as delivered in routine care. It may offer individuals who are underrepresented in treatment trials an opportunity to share their experiences. This interpretation is supported by the idea that individuals who claimed to have received CBT were more likely to post negative content than those who did not report undergoing CBT. These findings then suggest how to improve CBT in routine care.

Another explanation, not necessarily a competing one, is that social media facilitates or perhaps even promotes the dissemination of negative information [8] and misinformation [9] in regard to mental health treatments, as it does for other topics like politics. To take one example, CBT is among the few treatments with empirical support for PTSD [2], yet an individual relying on TikTok for information may falsely assume that there is a strong debate about the efficacy of CBT for PTSD. While there are a few on social media that are making these negative, potentially misinformed, posts about CBT, their voices are quite loud as evidenced by having a larger number of views and likes compared to positive/neutral videos, and generating more engagement in the form of comments. From this perspective, there is a need to combat negative public perceptions about CBT [10].

## Abbreviations

CBT: cognitive behavioral therapy
PTSD: posttraumatic stress disorder

## Data Availability

We share the data (ie, 200 video ratings) and the analyses on Open Science Framework [6].
